# Mesenchymal stromal cells for the treatment of Alzheimer’s disease: Strategies and limitations

**DOI:** 10.3389/fnmol.2022.1011225

**Published:** 2022-10-06

**Authors:** Shobha Regmi, Daniel Dan Liu, Michelle Shen, Bhavesh D. Kevadiya, Abantika Ganguly, Rosita Primavera, Shashank Chetty, Reza Yarani, Avnesh S. Thakor

**Affiliations:** Department of Radiology, Interventional Radiology Innovation at Stanford (IRIS), Stanford University, Palo Alto, CA, United States

**Keywords:** mesenchymal stromal cells, mesenchymal stem cells, Alzheimer’s disease, microglia, neurons, neuroprotection

## Abstract

Alzheimer’s disease (AD) is a major cause of age-related dementia and is characterized by progressive brain damage that gradually destroys memory and the ability to learn, which ultimately leads to the decline of a patient’s ability to perform daily activities. Although some of the pharmacological treatments of AD are available for symptomatic relief, they are not able to limit the progression of AD and have several side effects. Mesenchymal stem/stromal cells (MSCs) could be a potential therapeutic option for treating AD due to their immunomodulatory, anti-inflammatory, regenerative, antioxidant, anti-apoptotic, and neuroprotective effects. MSCs not only secret neuroprotective and anti-inflammatory factors to promote the survival of neurons, but they also transfer functional mitochondria and miRNAs to boost their bioenergetic profile as well as improve microglial clearance of accumulated protein aggregates. This review focuses on different clinical and preclinical studies using MSC as a therapy for treating AD, their outcomes, limitations and the strategies to potentiate their clinical translation.

## Introduction

### Alzheimer’s disease

Alzheimer’s disease (AD) is a neurodegenerative disease and the leading cause of age-associated dementia. It is the eighth leading cause of death in the United States and affects approximately 6.2 million people while accounting for about $305 billion in healthcare costs annually (Wong, 2020). AD is characterized by progressive brain damage that slowly destroys memory and the ability to learn, which eventually hinders patients in performing daily activities. Studies have identified three stages of AD progression: preclinical stage, mild cognitive impairment stage and dementia stage (No author list, 2021). The preclinical stage is of varied duration in which patients demonstrate no observable clinical symptoms but begin to exhibit early pathophysiological signs of AD, such as elevated amyloid-beta (Aβ) peptide and a reduction in glucose metabolism in the brain (No author list, 2021). However, while the brain can compensate for these early AD pathologies for an extent of time, the mild cognitive impairment stage is inevitably expressed as the disease progresses. Approximately one-third of patients with mild cognitive impairment then advance into the dementia stage within 5 years of symptom onset (No author list, 2021).

Alzheimer’s disease can be stratified into two main types: early onset and late-onset AD. Late-onset AD, which is mostly comprised of sporadic AD, is typically diagnosed at age 65 and older, and accounts for about 90% of the total AD cases (Reitz et al., 2020). On the other hand, early onset AD accounts for 5–10% of AD cases and is typically diagnosed before the age of 65 (Reitz et al., 2020). While most AD cases are sporadic, approximately 1% of cases are familial, meaning that the patient inherited AD-inducing genetic mutations from their parents (van der Flier et al., 2011). These familial cases usually manifest as early onset AD and are commonly associated with genetic mutations in amyloid precursor protein (APP), presenilin 1 (PS1), and presenilin 2 (PS2) (Swerdlow, 2007; van der Flier et al., 2011).

#### Pathophysiology

Although the precise pathophysiology of AD remains inconclusive, the progressive accumulation of Aβ plaques and neurofibrillary tangles (NFTs), which are aggregates of hyperphosphorylated tau protein, have been identified as the primary hallmark of AD. The extracellular deposition of Aβ plaques and the intracellular formation of NFTs leads to synapse loss, dystrophic neurites, microgliosis, and astrogliosis, all of which contribute to AD-associated brain atrophy (Pini et al., 2016). Interestingly, the formation of Aβ plaques is spatially and temporally separated from NFTs (Busche et al., 2019). The aggregation of Aβ plaques is an early event in the AD trajectory and is observed in the preclinical stage, while the accumulation of NFTs occurs at a timepoint closer to the later stages of AD, when neuronal dysfunction and degeneration has begun to induce clinical symptoms (He et al., 2018). Moreover, Aβ plaque accumulation initiates in the neocortex and progresses into deeper brain regions, while NFT accumulation initiates in the medial temporal lobe and spreads outward toward the Aβ-rich neocortex (Busche et al., 2019).

In AD, neuronal loss is found to be focal, predominantly in the cortex and hippocampus regions. Thus, it has been suggested that the coexistence of Aβ plaques and NFTs correlates with AD-driven behavioral symptoms such as memory loss and impaired cognitive abilities. The onset of these symptoms are likely a result of synapse loss, failure to maintain axon and dendrite functions, as well as extensive neuronal damage and death in the cortex and hippocampal regions (Bloom, 2014). Therefore, the transition from the asymptomatic preclinical stage into symptomatic stages of AD may be associated with the propagation of tau pathology into the Aβ plaque-rich cortex, suggesting a collaborative interaction between the two aggregates toward AD progression (Busche et al., 2019).

The aggregation of misfolded proteins, such as Aβ plaques and NFTs, is involved in the pathophysiology of various neurodegenerative diseases. Chaperone proteins, which influence protein folding, can contribute to both AD protection and advancement. For instance, heat shock proteins (HSPs), mainly HSP70 and HSP90, have been described as chaperone proteins that play critical roles in neurodegenerative diseases (Martin-Pena et al., 2018; Gupta et al., 2020). While HSP70 facilitates the clearing and refolding of misfolded proteins in AD, HSP90 stabilizes the misfolded proteins, leading to augmentation of Aβ aggregation and neurodegeneration (Gupta et al., 2020). Based on this hypothesis, HSP70 inducers and HSP90 inhibitors may have therapeutic potential in clearing tau and Aβ aggregates (Gupta et al., 2020).

Genetic mutations account for familial AD cases. Mutations in APP, PS1, and PS2 alter normal APP processing and produce the neurotoxic Aβ oligomer that gives rise to Aβ-induced neuro-toxicity and aggregation of Aβ plagues (Benilova et al., 2012). However, Aβ pathology is not the sole determinant of AD pathology, since disease onset is accompanied by the formation of NFTs in neurons. These intracellular aggregates are composed of hyperphosphorylated tau proteins and are associated with impaired microtubular cytoskeleton formation, synapse loss, and neurodegeneration (Alonso et al., 1996; Guillozet et al., 2003).

The interconnection between Aβ and tau pathology is demonstrated by the synergistic and correlative nature of amyloidosis and tau hyperphosphorylation, which are also interrelated in the induction of neural toxicity (Busche et al., 2019; Griner et al., 2019; Tripathi and Khan, 2020). An example of Aβ and tau interaction occurs in the mitochondria. Studies have shown that APP can be targeted to the mitochondria, and this mitochondria localization of APP correlates with AD adversity and only occurs in pathogenic brain areas of AD patients (Lin and Beal, 2006). The aggregated APP gets processed into the accumulative Aβ peptide by active γ-secretase complex, which leads to Aβ presence in the mitochondria. Aβ presence in the mitochondria then generates hyperphosphorylated tau proteins by interacting with mitochondrial Drp1 protein, which, in turn, disrupts microtubule function and induces neural toxicity (Manczak et al., 2011; Manczak and Reddy, 2012). Mitochondrial Aβ can also interact with Aβ–binding alcohol dehydrogenase (ABAD), leading to mitochondrial dysfunction and the production of reactive oxygen species (ROS) (Lin and Beal, 2006).

The main consequence of mitochondrial dysfunction, ROS accumulation and increased oxidative stress, serve as the major factors involved in AD pathogenesis. Postmortem studies in AD brains have found evidence of oxidative stress in the form of lipid peroxidation, DNA, RNA, and protein oxidation, and decreased antioxidant enzymes (Marcus et al., 1998; Pratico, 2008). This evidence supports the oxidative stress hypothesis as a significant mechanism of AD-induced neurodegeneration and neuron death (Marcus et al., 1998; Pratico, 2008). In fact, both Aβ aggregation and tau hyperphosphorylation can directly increase oxidative stress, since Aβ can act as an oxidant while hyperphosphorylated tau induces neuroinflammation and the consequent microglial ROS production (Nunomura et al., 2006; Alavi Naini and Soussi-Yanicostas, 2015; Cheignon et al., 2018).

Interestingly, postmortem analysis of AD brains from 20 AD patients also identified breakdown of the blood-brain barrier (BBB) (Nelson et al., 2016), likely due to pericyte detachment and degradation that is also observed during AD pathology. The loss of pericytes contribute to reduced BBB and dysregulated BBB transport, which increases the amount of vascular infiltrate entering the brain. Since soluble Aβ peptide is regularly transported across the BBB under normal physiological conditions, the disruption of BBB transport alters the balance between the efflux and influx of Aβ peptides, thereby contributing to reduced Aβ clearance, increased Aβ burden, and the formation of extracellular Aβ plaques in the brain (Deane et al., 2009). Downregulated glucose transport to the brain during AD progression also accelerates BBB breakdown and Aβ pathology, since the loss of glucose contributes to an energy deficiency that induces neuron necrosis and the resulting neuroinflammation (Nelson et al., 2016; Montagne et al., 2017).

Breakdown of the BBB leads to capillary leakage, which fosters neuronal death and injury *via* the accumulation of vascular-origin neurotoxic products in the brain. These neurotoxic products include RBC-derived hemoglobin, immunoglobulins, fibrinogen, thrombin, and plasminogen. Free Fe^2+^ from the vasculature can cause further ROS generation, and an increase in oxidative stress. In addition, the entry of albumin from the vasculature to the brain can inhibit blood flow, which contributes to ischemia or hypoxia-induced edema (Zlokovic, 2011). The BBB breakdown enhances neuroinflammation by enabling neutrophils and other immune cells to enter the brain (Zenaro et al., 2015). Enhanced neuroinflammation fosters neurotoxicity by producing ROS from activated immune cells, which causes synapse loss and neuronal damage and ultimately death (Schain and Kreisl, 2017). However, not all neuroinflammation is neurotoxic, and transient neuroinflammation and mobilization of blood-borne myeloid cells to the CNS during early stages of neural damage can have a neuroprotective effect. Alleviation of cognitive symptoms in an AD mouse model has been achieved by blockade of programmed cell death receptor 1 (PD-1/PD-L1), which induces a systemic immune response and enhances recruitment of monocyte-derived macrophages to the brain (Rosenzweig et al., 2019). However, even though immune enhancement achieved by checkpoint blockade has the potential to alleviate both Aβ and tau pathologies in some cases of AD, PD-1 blockade in another study failed to increase the infiltration of monocyte-derived macrophages into the brain and was unable to alter the Aβ burden (Baruch et al., 2016; Latta-Mahieu et al., 2018; Rosenzweig et al., 2019). Thus, additional neuroimmunology mechanisms and treatment options should be considered better to elucidate the role of neuroinflammation in AD pathology.

#### Current treatment approaches

There are currently two major approaches to AD intervention: symptomatic treatment, and disease-modifying therapy. These interventions are mainly recognized as pharmacological or cellular treatments.

##### Pharmacological treatments

Pharmacological AD treatment mainly utilizes psychotropic drugs that aim to alleviate behavioral and cognitive AD symptoms such as dementia. For instance, acetylcholinesterase (AChE) inhibitors, responsible for increasing the expression and half-life of the acetylcholine neurotransmitter, have been used to improve AD-induced cognitive function. AChE inhibitors such as rivastigmine, galantamine and donepezil, and NMDA-receptor antagonists like memantine, have been approved by the FDA for AD treatment (Alzheimer’s Association, 2019). However, these drugs are not able to limit the progression of AD and have several side effects. Thus, there is need for development of a molecule that can target multiple factors involved in AD.

Recently, therapies utilizing APP inhibitors, ROS inhibitors, anti-inflammatory, and anti-tau factors have been proposed as potential treatments for AD (Scarpini et al., 2003; Cummings et al., 2019; Xie et al., 2020). However, their efficacy remains uncertain, since some of them are still undergoing clinical trials while others have failed to demonstrate therapeutic efficacy in clinical settings. Recently, a monoclonal antibody targeted against Aβ (aducanumab) was approved for treating AD in the United States; however, the approval was highly controversial due to a lack of evidence that the drug is effective (Rabinovici, 2021).

##### Cell therapy

In contrast to the symptom-oriented approach of pharmacological therapies, cell therapy aims to target the origin of AD pathologies by replacing lost neurons, clearing toxic aggregates, stimulating neuronal precursors, and enhancing neuroprotection (Si and Wang, 2021). Cell therapy typically utilize stem cells, chosen for their multilineage differentiation and self-renewal capacities. Under the umbrella of the cell therapies are two main therapeutic strategies. The first seeks to directly supply new neurons *via* engraftment and differentiation of transplanted stem cells (i.e., cell replacement therapy). The second relies on the transplanted cells’ ability to release soluble factors that indirectly stimulate endogenous neural regeneration or promote neuroprotection. Various pre-clinical research and clinical trials have been conducted to determine the therapeutic efficacy of stem cell-based therapies for intractable neurodegenerative diseases like AD.

Different types of stem cells have been utilized for cell therapy in AD. Cell replacement therapies rely on transplanted cells engrafting and differentiating into neuronal fates, and thus must use competent cells to give rise to neurons. These include induced pluripotent stem cells (iPS), embryonic stem cells (ES), and neural stem cells (NSCs). However, ES and iPS cells have inherent risks of teratoma formation and immune rejection. Additionally, transplanted cells would have to demonstrate robust engraftment and site-appropriate neuronal differentiation and synaptic integration, which has not been achieved. Due to these difficulties, some have given up on direct engraftment of stem cells and have turned instead to the ability of adult stem cells to stimulate endogenous neural regeneration *via* paracrine effects. Mesenchymal stem/stromal cells (MSCs) have been a popular platform for cell therapy in general. While they are not competent to give rise to neural cell types by themselves, they have been described to have anti-inflammatory and immunomodulatory properties. Preclinical studies have reported the therapeutic efficacy of MSC-based therapies, and several clinical trials have explored the potential of clinically translating these therapies.

### Characteristics of mesenchymal stem/stromal cells

When they were first discovered, MSCs were recognized as a subcomponent of the bone marrow (BM) cell population. They were defined by the International Society of Cellular Therapy (ISCT) as adherent fibroblastic cells that can differentiate into osteocytes, chondrocytes, and adipocytes (Dominici et al., 2006). In addition, MSCs express cell surface markers such as CD105, CD73, and CD90, and do not express CD45, CD19, CD11b, and HLA-DR surface molecules (Dominici et al., 2006; Maleki et al., 2014). MSCs cannot be characterized by the expression of a single, specific marker, and they behave differently under different conditions like hypoxia and inflammation. The metabolic profile, secretomes, and proteome of MSCs may also vary based on their origin, culture conditions and microenvironment. These environment and origin-based variations make it challenging to identify and characterize MSCs, and the criteria provided by ISCT might not be enough for the proper characterization of MSCs (Maleki et al., 2014). Indeed, it is generally agreed that MSCs are not a coherent cell type, but rather a mix of various stem, progenitor, and mature cell types. Despite these issues, MSCs have remained popular for cellular therapy research, as they are easy to obtain and expand *in vitro*.

### Applications of mesenchymal stem/stromal cells and mesenchymal stem/stromal cell-derived therapeutics

Mesenchymal stem/stromal cells have been established as an important platform for cell therapy for treating various injuries and illnesses. Several clinical trials have been conducted to assess the therapeutic efficacy of MSC-derived therapies for a variety of diseases, but only 9 hMSC-based products have acquired legal approval for clinical application. In South Korea, Cellgram AMI, Cartistem, Cupistem, and Neuronata-R have been approved for the treatment of myocardial infarction, cartilage defects, Crohn’s diseases and amyotrophic lateral sclerosis, respectively. Similarly, Prochymal, an intravenous formulation of mesenchymal stem cells, has been approved in Canada, New Zealand, and Australia while TEMCELLS has been approved in Japan for the treatment of acute graft versus host disease (GVHD). Recently, STEMIRAC and Stempeucel have been approved for the treatment of spinal cord injury and Buerger’s disease-induced critical limb ischemia, respectively (Lopez-Beas et al., 2020). Some of these commercial MSC-based therapeutics have shown beneficial effects whereas the results of a few other products have not been published yet.

## Therapeutic potential of mesenchymal stem/stromal cells for AD treatment

Neurodegenerative diseases, like AD, are characterized by abnormal protein aggregation leading to neuroinflammation and neuronal cell damage. The neuronal loss leads to synaptic dysfunction that fosters clinical symptoms such as memory, cognitive, and behavioral impediments (No author list, 2021). MSC-based therapies have been investigated as a cell therapy for AD due the paracrine ability of MSCs to secrete growth factors, anti-inflammatory proteins, membrane receptors, and microRNAs (miRNA) that aid in the reduction of neuronal loss by blocking apoptosis and increasing neurogenesis, synaptogenesis, and angiogenesis (Nooshabadi et al., 2018).

**FIGURE 1:**
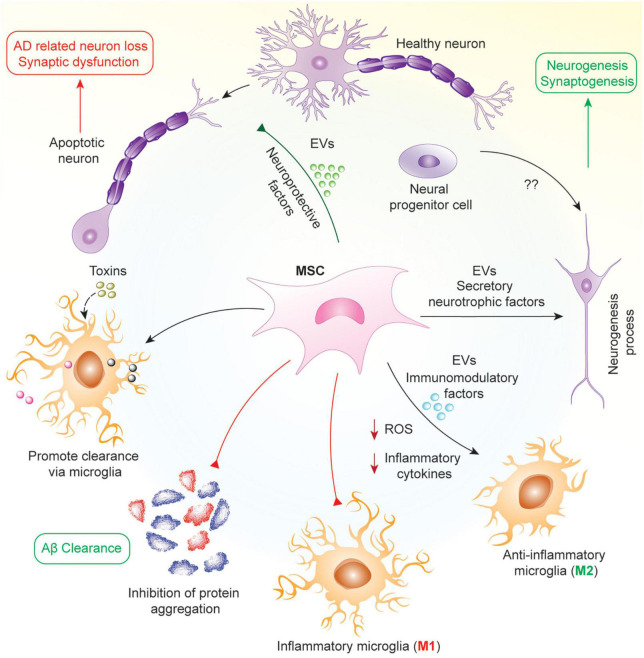
Mechanism of protective effects of MSCs in AD. In AD, neuronal loss and synaptic dysfunction occur due to the apoptosis of neurons by accumulating different proteins such as Aβ and hyperphosphorylated tau. MSCs work in pleiotropic mechanisms in this pathology to attenuate AD *via* secretion of various neuroprotective and neurotrophic factors such as VEGF, GDNF, BDNF, and several miRNAs in soluble or insoluble form as EVs. As a result, MSCs help in the clearance of aggregated proteins by increasing microglial phagocytosis, reprogramming microglia into anti-inflammatory phenotype, attenuating oxidative stress and neuronal apoptosis, and promoting neurogenesis from neural progenitor cells.

Mesenchymal stem/stromal cells are commonly derived from bone marrow and adipose tissues, and are capable of secreting factors that promote recruitment, proliferation and differentiation of other neural stem cells. Their role as an antioxidant coupled with their anti-apoptotic effects foster the inhibition of neuronal cell death, and secrets growth factors, such as brain-derived neurotrophic factor (BDNF) and glial cell line-derived neurotrophic factor (GDNF), to promote neurogenesis *via* the stimulation of neural progenitor cells (Harris et al., 2012; Van Velthoven et al., 2012; Reza-Zaldivar et al., 2018). In addition, MSCs exert immunomodulatory effects by inhibiting the activation of inflammatory microglia (M1) and promoting the activation of the anti-inflammatory microglia (M2) to prevent further tissue damage induced by chronic neuroinflammation. MSCs also accelerate the accumulation of microglia around Aβ deposits to promote Aβ clearance, and studies have revealed that MSCs can enhance activation of autophagy, which might be responsible for lysosomal clearance of Aβ plaques (Shin et al., 2014; Yokokawa et al., 2019; Figure 1).

In early stage AD, there is aggregation of Aβ and inefficient clearance of these aggregates resulting in the formation of amyloid plaques. In mid-stage AD, there is the formation of neuro-fibrillary tangles and neuronal cell death due to these aggregates. Finally, in late-stage AD, there is inflammation and oxidative stress mediated by reactive microglia. Hence, the mechanism by which MSCs work will be dependent on the stage of the disease in which they are being used. In early and mid-stages, MSCs will work to inhibit Aβ generation and promote its effective clearance, alter APP processing, decrease tau phosphorylation and increase proteasomal activity resulting in reduced accumulation of ubiquitin-conjugated proteins (Lee et al., 2017; Mendonça et al., 2019; Neves et al., 2021). In later stages, their effects will be more geared toward microglial reprograming, reducing the number of reactive microglia in the brain and promoting anti-inflammatory/anti-oxidant strategies (Cui et al., 2017; Zhao et al., 2018; Peruzzaro et al., 2019). Furthermore, MSCs have also been shown to promote microglia and autophagy mediated clearance of protein aggregates including Aβ (Kim et al., 2012; Shin et al., 2014; Yokokawa et al., 2019). Throughout all stages of AD, MSCs are able to protect neurons from cell death *via* secretion of different neuroprotective factors, growth factors and, mitochondrial transfer (Cui et al., 2017; Wang et al., 2018; Zhang et al., 2020). Hence, MSCs have multiple effects based on the disease condition, with their activity determined by the surrounding microenvironment in which they finally reside following their administration.

As stated earlier, the anti-inflammatory, immunomodulatory, and neuro-regenerative aspects of MSCs highlight them as potential therapeutics for AD. The therapeutic potential of MSCs has been suggested by various studies, in which intravenous injection of placenta-derived MSCs in Aβ-infused mice significantly inhibited neuroinflammation while improving cognitive function (Yun et al., 2013). The intravenous delivery of human umbilical cord mesenchymal stem cells (UC-MSCs) in transgenic AD mice (Tg2576) inhibited oxidative stress while fostering neuro-repair and neurogenesis (Cui et al., 2017). The therapeutic effect of UC-MSC engraftment was enhanced when UC-MSCs were treated with resveratrol, which is an activator that has been suggested to rejuvenate and improve the survival and differentiation of resident stem cells (Gorbunov et al., 2012; Wang et al., 2018). In addition, Aβ accumulation was significantly decreased by UC-MSC infusion (Lykhmus et al., 2019). Aβ deposition in the hippocampus and cortex regions of APP/PS1 mice was reduced after intra-carotid arterial injection of UC-MSCs, which is also associated with improved cognitive function *in vivo* (Boutajangout et al., 2017).

In addition to UC-MSCs, those from other sites of origin have also demonstrated therapeutic efficacy for AD. For instance, stereotactic injection of amniotic MSCs into bilateral hippocampi of APP/PS1 mice significantly stimulated microglial activation and phagocytic activity, resulting in Aβ clearance, neurogenesis and cognitive improvement (Zheng et al., 2017). Intracerebral injection of menstrual blood-derived MSCs correlates with reduced BACE1 (β-APP cleaving enzyme 1) and β-CTF in the cortex and hippocampus of APP/PS1 mice, suggesting an inhibition of β-secretase activity that diminished the formation of Aβ plagues (Zhao et al., 2018). Preclinical studies on the effectiveness of MSC-derived treatments in AD are listed in Table 1.

**TABLE 1:** Application of MSCs for Alzheimer’s disease in preclinical models.

Source	Dose	Route	Animal model	Major targets	Results	Reference
Human placental MSCs	1 × 10^5^, 5 × 10^5^, and 1 × 10^6^	I.V.	Aβ_1–42_ infused mouse	APP, BACE1, Aβ, β-secretase, and γ-secretase activity	Improved cognitive function (MWM); decreased Aβ, APP, and BACE levels; decreased astrocyte and microglia area; decreased expression of iNOS, COX2; decreased hippocampal apoptosis; increased hippocampal neurogenesis (DCX^+^)	Yun et al., 2013
Human UC-MSCs	1 × 10^6^	I.V.	STZ-induced rats	Reactive microglia	Improved cognitive function (BMT, OFT, and MBT); restored hippocampal volume and neuron density; no restoration of hippocampal neurogenesis (DCX^+^); no change in astrocyte or microglia area; the decreased proportion of reactive microglia (by morphology); modestly increased expression of some synaptic proteins (SYT1, SYP, and GAD65).	Villar et al., 2020
Rat AD-MSCs primed with melatonin (MT)	1 × 10^6^	I.V.	Aβ-injected rat model	Aβ clearance	Improved cognitive function (NORT, OFT, EPM, PAL, and MWM), melatonin priming further improved performance on some tests (NORT, MWM, and PAL); decreased Aβ deposits; decreased microglial density.	Nasiri et al., 2019
Human UC-MSCs	2 × 10^6^	I.V.	Tg2576 mice	Oxidative stress	Improved cognitive function (MWM); no change in hippocampal Aβ; decreased oxidative stress; increased expression of BDNF, Sirt1, and SYN in the hippocampus.	Cui et al., 2017
Human UC-MSCs, plus oral administration of resveratrol	1 × 10^6^ administered every 2 weeks for 2 months	I.V.	Tg2576 mice	Oxidative stress and senescence	Increased hippocampal engraftment with resveratrol (3-fold); improved cognitive function (MWM); decreased hippocampal apoptosis; increased hippocampal neurogenesis (Nestin+/βIII tubulin^+^) and neuron density; increased expression of BDNF, NGF, NT-3, and Sirt1 in the hippocampus; effects generally enhanced with resveratrol.	Wang et al., 2018
Human UC-MSCs, mouse placental MSCs	1 × 10^6^	I.V.	LPS-induced mice	nicotinic acetylcholine receptors, Aβ accumulation, mitochondria	Improved cognitive function (NORT); decreased Aβ levels; qualitatively increased numbers of microglia and astrocytes.	Lykhmus et al., 2019
Human amniotic MSCs	1 × 10^6^	Intracerebral (bilateral hippocampus)	APP/PS1 mice	Aβ plague clearance	Improved cognitive function (MWM); reduced Aβ deposits; increased microglia area and activation (ED1^+^); decreased expression of TNF-α, IL-1β, increased expression of IL-10, TGF-β in the brain; increased expression of IDE, NEP, BDNF; increased hippocampal neurogenesis (DCX^+^, BrdU^+^/NeuN^+^); increased dendritic spine density in cortical and hippocampal neurons.	Zheng et al., 2017
CM of rat AD-MSCs cultured in hypoxic condition	200 μL CM administered eight times at 1-day intervals	Intraperitoneal	Intra-hippocampal Aβ_1–40_ injected rats	Neurotrophic and anti-inflammatory factors	Improved cognitive function (MWM and NORT); increased hippocampal neuron density; decreased hippocampal Aβ deposits; decreased expression of TLR2, TLR4, IL-1β, and TNF-α	Mehrabadi et al., 2020
Human UC-MSCs	1 × 10^6^	I.V. and I.C.V.	Intra-hippocampal Aβ_1–42_ injected rats	Promoting cholinergic function of neurons	Marginally improved homing to hippocampus with magnetic guidance (non-significant); improved cognitive function (PAL and MWM); increased expression of AChE and ChAT in the hippocampus; effects increased with magnetic guidance.	Hour et al., 2020
Rat BM-MSCs	3 × 10^5^	I.V.	APP/PS1 mice	Microglia and oxidative stress	Marginally improved cognitive function (MWM); decreased oxidative stress (EPR imaging); decreased Aβ deposits; marginally decreased soluble Aβ but no change in insoluble Aβ; decreased microglia area in cortex; marginal increase in number of associated microglia per Aβ plaque; increased proportion of CD14^+^ microglia.	Yokokawa et al., 2019
MSCs, MSC-derived EVs (host species and tissue of origin not reported)	1 × 10^6^ or 10 μg exosomes	Intracerebral (bilateral hippocampus)	Intra-hippocampal Aβ_1–42_ injected mice	Neurons and neuro-regeneration	Improved cognitive function (MWM and NORT); increased SVZ neurogenesis (PSA-NCAM^+^/DCX^+^); comparable results between MSCs and MSC-derived EVs.	Reza-Zaldivar et al., 2019
Human UC-MSCs	5 × 10^5^	Intra-arterial (carotid) with BBB opening (mannitol)	APP/PS1 mice	Aβ burden	Improved motor function (rotarod) and cognitive function (NORT); decreased Aβ deposits in motor cortex and hippocampus; no change in astrocyte area; decreased microglia area.	Boutajangout et al., 2017
Human UC-MSCs	2 × 10^6^	I.V.	APP/PS1 mice	Aβ and neuroinflammation	Improved cognitive function (MWM); decreased Aβ deposits; increased microglia area on day one but decreased microglia area on day four; increased IL-10, decreased IL-1β, and TNF-α.	Xie et al., 2016
Human UC-MSCs, primed with Aβ	1 × 10^5^	I.C.V.	5XFAD mice	TGF-β mediated neuroprotection and Aβ clearance	Reduced cell death; reduced Aβ deposition; more significant effect in primed MSCs.	Park et al., 2021
Human UC-MSCs	1 × 10^5^	I.C.V.	5XFAD mice	GAL-3-mediated inhibition of GSK-3β targeting tau	Improved cognitive function (OFT, T-maze); decreased tau hyperphosphorylation, possibly through GAL-3-mediated inhibition of GSK-3β	Lim et al., 2020
Human UC-MSCs	1 × 10^5^, administered once, or three times at 4-week intervals	Intrathecal	APP/PS1 mice	Aβ mediated neuronal loss	Increased hippocampal neurogenesis (SOX2^+^GFAP^+^ and NeuN^+^); reduced Aβ levels, possibly through GDF-15	Kim H. J. et al., 2015
Human UC-MSCs	1 × 10^5^	I.C.V.	5XFAD mice	TSP-1 mediated neuroprotection	Alleviation of hippocampal synapse loss, possibly through TSP-1	Kim et al., 2018b
Rat BM-MSCs	1 × 10^5^	I.C.V.	APP/PS1 mice	miR-146a targeting astrocytes for improving synaptogenesis	Improved cognitive function (MWM), no change in hippocampal Aβ deposition, neuron count, or synapse density, a slight decrease in activated microglia, and decreased TNF-α possibly through miR-146a-mediated suppression of NF-κB.	Nakano et al., 2020
Human BM-MSCs	2 × 10^6^	I.V.	APP/PS1 mice	Aβ-mediated inflammation	Improved cognitive function (MWM); decreased Aβ and BACE, increased A2M levels in brain; decreased serum IL-1, IL-2, TNF-α, and IFN-γ.	Wei et al., 2018
Mouse BM-MSCs	1 × 10^6^	I.V.	APP/PS1/Tau 3×Tg mice	Migration of MSCs to brain	Higher MSC migration to brain in AD mice (0.31%) compared to control mice (0.21%), but extremely low in both cases	Park et al., 2018
Mouse BM-MSCs	1 × 10^6^	I.V.	APP/PS1 mice	Aβ pathology	No change in Aβ plaque numbers; slight reduction in plaque size in hippocampus but not cortex; slight decrease in microglia count in the cortex; reduced microglia size; decreased expression of TNF-α, IL-6, MCP-1, and NGF in brain, though not consistent by region; no change in expression of IL-10, CCR5, BDNF, VEGF, and IFN-γ.	Naaldijk et al., 2017
CM of mouse BM-MSCs primed with AD mouse brain homogenate	CM of 1 × 10^6^ cells cultured for 24 h, single or repeated dose	I.V. or intranasal	APP/PS1	Aβ-mediated inflammation and neuronal loss	Improved memory function (NORT); decreased Aβ deposits; decreased microglia and astrocyte area; decreased astrocytic TNF-α expression; increased neuron density in cortex (+10%) and hippocampus (+25%); decreased hippocampal atrophy; increased survival (+45%)	Santamaria et al., 2021
EVs derived from cytokine-primed human BM-MSCs	1.5 × 10^10^ EVs (30 μg), administered twice at 18-h interval	Intranasal	APP/PS1/Tau 3×Tg mice	Aβ mediated activation of reactive microglia	Reduced microglia density, size, and activation (CD68^+^); increased dendritic spine density in cortical and hippocampal neurons.	Losurdo et al., 2020
RVG-tagged EVs derived from mouse BM-MSCs	5 × 10^11^ EVs	I.V.	APP/PS1 mice	Improving migration to brain for anti-inflammatory effect	Improved homing to the brain; decreased Aβ deposits; decreased astrocyte area; improved cognitive function (MWM); decreased expression of TNF-α, IL-1β, and IL-6, increased expression of IL-4, IL-10, and IL-13 in the brain.	Cui et al., 2019
Mouse BM-MSCs overexpressing CX3CL1 and Wnt3a	4 × 10^5^	I.C.V.	APP/PS1 mice	Aβ, synaptic loss and neurodegeneration and neuroinflammation	With CX3CL1 overexpression: decreased microglia frequency, decreased TNF-α and increased IL-10 expression in brain, increased expression of synaptic proteins (PSD95 and SYP) in cortex and hippocampus, no increase in cognitive function (MWM); with CX3CL1 and Wnt3a overexpression: increased cognitive function (MWM), increased neurogenesis in hippocampus (SOX2^+^Ki67^+^ and DCX^+^), increased phosphorylation of PI3K, Akt, and GSK3β (ser9)	Li et al., 2020
Mouse BM-MSCs overexpressing VEGF	1 × 10^6^	I.C.V.	APP/PS1 mice	Aβ mediated neuroinflammation	Improve hippocampal neovascularization, diminish senile plaques, inhibit inflammation, and rescue behavioral and cognitive functions as compared to control MSCs	Garcia et al., 2014
Human menstrual blood-derived MSCs	1 × 10^5^	Intracerebral (bilateral hippocampus)	APP/PS1 mice	Tau and Aβ mediated neuroinflammation	Improved cognitive function (MWM); decreased Aβ plaque area and tau hyperphosphorylation; decreased expression of β-CTF, BACE1, increased expression of IDE, NEP in the brain; increased microglial area and shift toward alternative phenotype.	Zhao et al., 2018
AD-MSCs	1 × 10^5^	stereotaxic surgery	APP/PS1 mice	Aβ mediated oxidative stress	Inhibition of oxidative stress and promotion of neurogenesis in hippocampus,	Yan et al., 2014b
Rat AD-MSCs	1 × 10^5^	Intracerebral (bilateral hippocampus)	APP/PS1 mice	Aβ mediated oxidative stress	Slightly improved memory function (NORT); decreased oxidative stress; increased neurogenesis (BrdU^+^/DCX^+^ cells) in hippocampus and SVZ.	
Human UC-MSCs	2 × 10^5^	Intracerebral (left hippocampus)	5XFAD mice	Proteasome activity	Increased proteasome activity *via* AgRP, reduced accumulation of ubiquitin-conjugated proteins.	Lee et al., 2017
Mouse BM-MSCs	1 × 10^6^	I.V., single or quadruple dose	APP/PS1/Tau 3×Tg mice	Tau and Aβ mediated neuroinflammation	Decreased neuroinflammation, altered APP processing, no decrease in Aβ plaques, decrease in tau phosphorylation	Neves et al., 2021

Unless otherwise noted, results describing microglia and astrocytes refer to positive staining for Iba1 and GFAP. BMT, Barnes maze test; EMT, elevated plus maze; I.C.V., intracerebroventricular; I.V., intravenous; MBT, marble burying test; MWM, Morris Water Maze; NORT, novel object recognition task; OFT, open field test; PAL, passive avoidance learning; CM, condition media; ADMSC, adipose derived mesenchymal stem cells; BM-MSC, bone marrow derived mesenchymal stem cells.

Despite the effectiveness of MSC-based therapies in preclinical studies, they have largely failed to be clinically translated. Although MSC therapies are feasible, safe, and well tolerated when delivered into the brain *via* stereotactic injection, a recent clinical trial found minimal therapeutic improvements in a 24-month follow-up period (Kim H. J. et al., 2015). Nevertheless, this phase 1 study paved way for further evaluation of MSC therapies in larger cohorts with long-term follow-up, and many clinical trials are currently being conducted in AD patients. In another clinical trial, a single dose I.V. injection of MSCs showed improvement in inflammation and neurocognitive function compared to a placebo (Oliva et al., 2021; Brody et al., 2022; Table 2). The dose of MSCs *via* stereotactic injection into the hippocampus was 3-to-33 times less than the clinical trial with I.V. injection. Both of these clinical trials support the potential of MSCs in treating AD. However, the variation in doses, MSCs source, disease onset time, route of delivery, and small study population hinder any definitive conclusions. Nevertheless, local injection of MSCs might be an effective strategy in decreasing the dose of MSCs being used for therapy due to minimal loss in the systemic circulation.

**TABLE 2:** Use of MSCs in Alzheimer’s disease in different clinical trials.

Source	Dose	Route	Study phase	Participants	Results	Study location	Clinical trial.gov ID
Lomecel-B (allogeneic BM-MSCs)	2 × 10^7^, or 1 × 10^8^ cells	I.V.	Phase1a, randomized, placebo-controlled study	33	Safe and well tolerated, effective in increasing anti-inflammatory and pro-vascular biomarker in serum, improve neurocognition and quality of life in treated patients compared to placebo	United States	(NCT02600130) (Oliva et al., 2021; Brody et al., 2022)
Autologous ADMSCs	2 × 10^8^ cells (9 times at 2 weeks intervals)	I.V.	1/2, randomized, double-blind, placebo-controlled study	21	−	United States	NCT03117738
Autologous ADMSCs	2 × 10^8^ cells (4 times at 2 weeks intervals)	I.V.	1/2a, open-label, non-randomized study	24	Withdrawn due to COVID-19 pandemic	United States	NCT04228666
Allogeneic BM-MSCs	Placebo or 1.5 × 10^6^cells/kg body weight	I.V.	2a, randomized, single-blind, placebo-controlled, crossover, multicenter study	40	−	United States	NCT02833792
Allogeneic UC-MSCs	1 × 10^7^ or 3 × 10^7^ cells administered 3 times at 4-week intervals	I.C.V.	1/2a, double-blind, single-center study	45	−	South Korea	NCT02054208 NCT03172117
Allogeneic human UC-MSCs	Placebo or 2 × 10^7^ cells, administered 8 times at 2-week intervals	I.V.	1/2, randomized, double-blind, placebo-controlled, multicenter study	16	−	China	NCT02672306
Allogeneic UC-MSCs	Placebo, 3 × 10^6^, or 6 × 10^6^ cells per brain	Intra hippocampus bilaterally and right precuneus	Phase 1, open-label, single-center study	9	Feasible, safe, and well-tolerate with no effect on AD pathophysiology.	South Korea	NCT01297218 NCT01696591 (Kim H. J. et al., 2015)
Autologous human ADMSCs	N/A	N/A	N/A	1	−	United States	NCT04855955
Allogeneic human UC-MSCs	1 × 10^8^ cells, administered 4 times at 13-week intervals	I.V.	1, prospective open-label study	6	−	United States	NCT04040348
Allogeneic human ADMSC-derived EVs	5, 10, or 20 μg EVs, administered twice a week for 12 weeks	Nasal drip infusion	1/2, open-label, single-center study	9	−	China	NCT04388982
Human P-MSCs	Placebo or 2 × 10^8^ cells, 1–2 times at 4-week interval	I.V.	1/2a, randomized, double-blind, placebo-controlled study	24	−	South Korea	NCT02899091
Autologous ADMSCs	Placebo or 2 × 10^8^ cells, administered 4 times at 4-week intervals	I.V.	2b, randomized, double-blind study	80	−	United States	NCT04482413
Allogeneic human UC-MSCs	2 × 10^7^ cells, administered 8 times at 2-week intervals	I.V.	1/2, open-label, single-center study	30	−	China	NCT01547689

## Impact of mesenchymal stem/stromal cells on neurons and neural progenitor cells

Mesenchymal stem/stromal cells are involved in neuroprotection *via* the secretion of anti-inflammatory cytokines and anti-apoptotic, angiogenic, and neurotrophic factors. Direct contact between primary neurons and MSCs is believed to enhance the long-term survival of neurons and may play a vital role in neuronal maturation and differentiation (Scuteri et al., 2006). Furthermore, MSCs have been shown to prevent oxidative stress in neurons as well as Aβ/tau-induced toxicity by clearing Aβ or tau *via* autophagy activation, increasing proteasome activity and promoting microglial phagocytosis (Yan et al., 2014b; Lee et al., 2017; Zhao et al., 2018; Neves et al., 2021; Santamaria et al., 2021). It had been postulated that MSCs could transdifferentiate into neuron-like cells, which would serve as an asset in AD therapies (Yang et al., 2013). The differentiation of MSCs into a neuronal lineage is still controversial, even though some research shows the ability of MSCs to differentiate into neural-like cells (Scuteri et al., 2011a; Divya et al., 2012; Jeong et al., 2013; Feng et al., 2014; Haragopal et al., 2015; Joe et al., 2015; Hernández et al., 2020; Zhou et al., 2020). Interestingly, a study by Lu et al. showed morphological and immunocytochemical changes in MSCs after their exposure to a medium for neuronal induction and differentiation, which they attributed to not be the consequence of true neuronal differentiation but rather a biological response to chemical stress (Lu et al., 2004). In another study, it was shown that newly generated neuronal cells regained their MSC morphology as soon as the neuronal induction therapy stopped, indicating that this change is reversible in many instances (Lukomska et al., 2019). Other studies have also shown a beneficial interaction between MSCs and NSCs in promoting neurogenesis (i.e., differentiation, proliferation and survival of NSCs), neuroprotection as well as facilitating the differentiation of MSCs to neural cells (Alexanian, 2005; Oh et al., 2011; Haragopal et al., 2015; Ju et al., 2015; Kim D. H. et al., 2015). Hence, the co-transplantation of NSCs and MSCs. or transplantation of NSCs-primed MSCs. could be an effective strategy for neuroregeneration and neuroprotection.

### Neuroprotective effect of mesenchymal stem/stromal cells on co-cultured neurons

Co-culturing MSCs with primary rat cortical neurons has been shown to protect neurons from apoptosis and enhance their survival (Scuteri et al., 2011b; Scheibe et al., 2012). MSC-co-cultured neurons demonstrated no signs of degeneration and survived 60 days or more, while primary neuronal cultures without MSC exposure experienced cellular death in a few days (Scuteri et al., 2011b). This MSC-induced neuroprotection and promotion of long-term neuron survival was suggested to be the result of downregulated matrix metalloproteinase (MMP) activity and the fostering of neuronal maturation (Scuteri et al., 2006, 2011b).

Co-culture of MSCs with hippocampal neurons in a transwell system protected neurons from Aβ-induced oxidative stress and prevented the synapse loss that is typically associated with Aβ exposure (de Godoy et al., 2018). This protective effect was speculated to be a consequence of MSC-mediated Aβ clearance (de Godoy et al., 2018). The study further reported that MSCs are capable of internalizing fibrillary Aβ and neurotoxic Aβ oligomers without compromising their own viability, proliferation and ROS levels, and that the internalized Aβ oligomers and fibrils then undergo endosomal and lysosomal clearance (de Godoy et al., 2018). This MSC-induced autophagy and lysosomal clearance correlates with neuroprotection *in vivo* and *in vitro* and insinuates a connection between MSCs and microglial activation (Shin et al., 2014). For instance, when LPS-stimulated microglia and dopaminergic neurons were co-cultured with MSCs, the anti-inflammatory and clearance-inducing effects of MSC led to a decrease in LPS-induced damage and suppression of dopaminergic neuronal loss (Kim et al., 2009).

### Role of mesenchymal stem/stromal cell-secreted neuroprotective factors

Mesenchymal stem/stromal cell-induced neuron survival and neuroprotection have been suggested to be a result of various MSC-secreted factors. For instance, thrombospondin-1 secreted by UC-MSCs has been shown to rescue synaptic dysfunction induced by Aβ deposition in hippocampal neurons *via* the upregulation of neuroligin-1 (NLGN1) and the voltage-activated Ca2+ channel subunit α2δ-1, both of which are involved in glutamatergic synapses and mediation of long-term potentiation (Kim et al., 2018b). In addition, MSCs have been shown to secrete BDNF growth factor to upregulate AKT phosphorylation while downregulating p38 phosphorylation in order to protect neurons against trophic factor withdrawal and ROS exposure (Wilkins et al., 2009). BDNF-overexpressing hMSCs have been shown to have an augmented neuroprotective effect (Scheper et al., 2019).

Other MSC-secreted neuroprotective factors include the antioxidant enzyme superoxide dismutase 3 (SOD3) and agouti-related peptide (AgRP) (Kemp et al., 2010). Inflammatory cytokines, specifically tumor necrosis factor alpha (TNF-α) and interferon-gamma (IFN-γ), can induce MSCs to secrete SOD3, thereby reducing the build-up of excess superoxide and enhancing neuronal and axonal survival *in vitro* (Kemp et al., 2010). Pretreating MSCs with the antioxidant tanshinone has been reported to reduce neuroinflammation and suppress ROS in rats with Aβ-induced neuroinflammation (Huang et al., 2019). In another study, when SH-SY5Y neuroblastoma cells were co-cultured with MSCs, the ubiquitin proteasomal system was significantly upregulated in a dose-dependent manner based on the concentration of the MSC-secreted cytokine AgRP (Lee et al., 2017). This upregulation in proteasome activity fosters the formation of autolysosomes, which propels the clearance of abnormal protein aggregates, enhances neuron survival and reduces cognitive impairment in AD (Lee et al., 2017).

### Neuroprotective effects of mesenchymal stem/stromal cell-secreted extracellular vesicles

The neuroprotective effect of MSCs is also exerted by secreted extracellular vesicles (EVs), which deliver antioxidant catalase and other MSC-derived factors (de Godoy et al., 2018). miRNA-21 derived from MSC-secreted exosomes has been shown to inhibit neuron apoptosis by downregulating the expression of phosphatase and tensin homolog (PTEN) and programmed cell death protein 4 (PDCD4) in rats with spinal cord injury (Kang et al., 2019). Interestingly, the secretome of MSCs varies based on the time that the conditioned media (CM) is collected, exerting different effects on neurons and glial cells. The CM collected during earlier time points (24 h) enhanced neuron viability while CM collected at later time points (96 h) contributed to higher glial viability (Ribeiro et al., 2011), though the factors responsible for this difference were not determined. Exosomal miR-21 from MSCs have been shown to not only inhibit neuronal apoptosis but also improve the cognition and memory in transgenic APP/PS1 mice, thereby reducing Aβ deposition and downregulating pro-inflammatory cytokines. This effect was further improved with exosomes from hypoxia pre-conditioned MSCs (Cui et al., 2018). miR-146a in BM-MSC derived exosomes decreased NF-kB in astrocytes thereby restoring their function, which, in turn, promoted synaptogenesis and improved cognitive function (Nakano et al., 2020). Recently, BM-MSC derived EVs exhibited a protective effect on hippocampal neurons by decreasing amyloid beta deposition and reducing inflammatory cytokines in an amyloid beta-induced rat model of AD; these effects were deeded to be mediated by miR-29c-3p which was shown to activate the Wnt/β-catenin pathway (Sha et al., 2021). In another study, miR-29b overexpressed exosomes from MSCs reduced neuronal cell death and the pathology of Aβ in a rat model of AD while also showing improvement in spatial learning and memory (Jahangard et al., 2020). Similarly, miR-455-3p from BM-MSCs also ameliorated neuronal injury in the hippocampus (Gan and Ouyang, 2022).

## Role of mesenchymal stem/stromal cells on microglia

Microglia are the resident phagocytic immune cells of the central nervous system (CNS). They are involved in immune surveillance, pathogen defense, and maintenance of homeostasis in the brain (Li and Barres, 2018). Microglia are also crucial for the development of the brain and play a critical role in neurogenesis, myelin turnover, and the modeling and pruning of synaptic architecture and network (Li and Barres, 2018).

Under normal brain physiology, microglia are responsible for the clearance and degradation of extracellular Aβ, which they recognize through microglial integrin receptors and recruitment of several enzymes such as the pro-inflammatory matrix metalloproteinases (MMPs) to restrict the formation of Aβ plaques (Konnecke and Bechmann, 2013; Doens and Fernandez, 2014; Hansen et al., 2018). However, in AD pathology, the accumulation of Aβ aggregates and other chemokines released from injured neurons prolongs the activation of microglia, which can lead to chronic neuroinflammation and subsequent accumulation of ROS (Leng and Edison, 2021). Activated microglia are characterized by elevated expressions of CD86, CD40 and Iba-1, and are also responsible for secretion of inflammatory cytokines and chemokines to recruit peripheral immune cells into the brain (Leng and Edison, 2021). However, while transient neuroinflammation is beneficial for neurogenesis and aggregate clearance, excessive or chronic neuroinflammation that fails to resolve itself can worsen AD symptoms due to ROS generation, necrosis, and collateral tissue damage in the brain (DiSabato et al., 2016; Schain and Kreisl, 2017; Yong et al., 2019). Thus, microglial activation plays a critical role in AD pathology and has been proposed as a target for AD therapy (Solito and Sastre, 2012; Siskova and Tremblay, 2013).

Mesenchymal stem/stromal cells have been proposed as mediators of AD therapy by targeting microglia due to their immunomodulatory effects. As a result, interactions between MSCs and microglia have been studied extensively in the context of neurological diseases. Intraparenchymal MSC transplantation in rats with traumatic brain injury has been shown to improve fine motor function, an effect the authors attribute to MSCs shifting microglia from a classical inflammation (CD86) to an alternative inflammation state (CD163) (Peruzzaro et al., 2019). MSCs transplanted in AD mice have been reported to reduce microglial production of pro-inflammatory factors TNF-α, IL-1β, iNOS, and COX-2, and upregulate the expression of Aβ-degrading enzymes such as insulin-degrading enzyme (IDE) and neprilysin (NEP) (Zhao et al., 2018). This led to an improved clearance of abnormal protein aggregates, including Aβ plaques and hyperphosphorylated tau, without causing chronic neuroinflammation (Zhao et al., 2018).

Interestingly, studies suggest that MSCs have the ability to reprogram microglia into an “M2-like” phenotype that is characterized by an increase in phagocytic activity and a reduction in neuroinflammation. In fact, MSCs induce a mixed microglial phenotype that is CD206-high, Arg1-high, CD86-high, IL-10-high, MCP-1/CCL2-high, PGE2-high, IL-1β-moderate, TNF-α-low, and NALP-3-low (Hegyi et al., 2014). Thus, it has been speculated that the therapeutic effect of MSCs in AD pathology is related to their ability to alter microglia cells from the inflammatory, neurotoxic phenotype to a neuroprotective, anti-inflammatory phenotype that fosters neuro-regeneration and repair (Hegyi et al., 2014).

### Impact of mesenchymal stem/stromal cell-secreted factors on microglia

The therapeutic benefits and tissue repair observed in MSC transplantation has been associated with their ability to modulate the functional behavior of cells in the brain *via* paracrine mechanisms associated with MSC-secreted cytokines, growth factors, chemokines, and EVs (Gnecchi et al., 2016). For instance, human UC-MSCs secrete soluble intercellular adhesion molecule-1 (sICAM-1) after co-culturing with BV2 microglia which, in turn, diminish accumulation of Aβ plaques (Kim et al., 2012). Release of sICAM-1 increased after Aβ induction in the BV2 microglia, and an up-regulation of NEP enzyme was observed in co-cultured microglia as compared to those that were not exposed to MSCs (Kim et al., 2012). sICAM-1 also interrupts CD40/CD40L activity, thereby reducing pro-inflammatory signaling and enhancing microglial phagocytosis for Aβ and tau deposits (Kim et al., 2012). In addition, MSCs also up-regulate the expression of CD14, an important receptor for Aβ uptake, in microglia to facilitate microglial internalization and clearance of Aβ deposits, both *in vitro* and *in vivo* (Yokokawa et al., 2019). Growth differentiation factor-15 (GDF-15) secreted by UC-MSCs has also been associated with enhanced BV2 microglial Aβ clearance, *in vitro* and *in vivo*, through the upregulation of IDE (Kim et al., 2018a).

The ability of MSCs to regulate microglia activation also relies on its secreted paracrine factors. For instance, transwell co-culture of MSCs and LPS-stimulated microglia attenuated the activation of microglia with increased IL-6, IL-10, and TGF-β expressions, and reduced NO and TNF-α production (Kim et al., 2009). MSC-secreted CX3CL1 also exerts a regulatory effect on microglia by inhibiting expression of TNF-α, inducible nitric oxide synthase (iNOS), and ROS (Giunti et al., 2012). This enables an alternate microglial activation that enhances microglial phagocytic capacity without the onset of neurotoxic, chronic neuroinflammation, thereby reducing cellular damage and apoptosis (Giunti et al., 2012). Thus, it has been suggested that the ability of MSCs to improve microglial phagocytic activity under the anti-inflammatory, neuroprotective phenotype is dependent on the secretion of CX3CL1 (Giunti et al., 2012). While MSCs induce functional changes to microglia, they do not appear to promote microglial proliferation (Giunti et al., 2012). In fact, studies suggest that MSCs actually exhibit anti-proliferative effect toward BV2 microglia by reducing TNF-α expression, increasing the percentage of BV2 microglia that are under G0/G1 cell cycle arrest even in the face of LPS stimulation (Jose et al., 2014). Table 3 summarize different studies showing the effect of MSCs in modulating microglia for decreasing neuroinflammation and neuroprotection.

**TABLE 3:** Studies exploring factors governing the neuroprotective/immunomodulatory effect of MSCs.

Source	Microglia cell type	Factors secreted	Effect of MSCs on microglia	References
hUC-MSCs	BV2 mouse microglia	sICAM	Enhanced NEP expression, reduced CD40 expression, increased microglial Aβ clearance	Kim et al., 2012
Rat MSCs	Rat primary microglia	TGF-β	Enhanced anti-inflammatory phenotype and phagocytic activity of microglia is mediated *via* TGF-β signaling	Noh et al., 2016
Mouse BM-MSCs	BV2 mouse microglia	TSG-6	Inhibited pro-inflammatory factors in TSG-6 dependent mechanism where NF-κB and MAPK signaling are inhibited in LPS-induced microglia	Liu et al., 2014
UC-MSCs	BV2 mouse microglia	GDF-15	Increased in insulin-degrading enzyme expression in microglia- mediated Aβ clearance *via* GDF-15 secretion from MSCs	Kim et al., 2018a
BM-MSCs	BV2 mouse microglia	Soluble CCL5	Promoted alternative activation of microglia and reduced in Aβ *via* neprilysin and interleukin-4 from alternatively activated microglia	Lee et al., 2012
Human amniotic-derived MSCs	BV2 mouse microglia	Nitric oxide	Decrease viability, migration of microglia and promoted anti-inflammatory phenotype of microglia	Yan et al., 2014a

## Limitations of mesenchymal stem/stromal cells for the treatment of Alzheimer’s disease

The therapeutic efficacy of MSC-based therapies depends on a variety of factors, including a homogenous cell population, the source of MSCs, the optimal dose of transplanted cells, time of transplantation, and suitable route for cell delivery. However, an optimal protocol for the isolation, characterization, and expansion of MSCs remains poorly characterized, and there is an unmet need to determine the appropriate dose, route and time for MSC transplantation. The occurrence of immune responses, especially from allogeneic transplantations of cells, also complicates the application of MSC therapies. While some clinical trials reported evidence of therapeutic efficacy, many studies failed to observe clinical improvement after MSC therapy. In addition to the above-mentioned variables, the lack of homing of MSCs to brain is another hurdle in the treatment of neurodegenerative diseases as blood-brain barrier (BBB) also increases the difficulty of MSC delivery to the brain.

## Strategies to overcome limitations

### Facilitating mesenchymal stem/stromal cell delivery to the brain

Although MSCs possess some homing capacity to sites of injury, very few intravenously injected cells successfully migrate to the target site, and the majority end up entrapped in the lung microvasculature instead of the brain (Ullah et al., 2019). The BBB is another major limiting factor that compromises the delivery of therapeutics, including MSCs, into the brain for treatment of neurodegenerative diseases (Hour et al., 2020). As a result, several studies have attempted to bypass the BBB by delivering MSCs *via* intraparenchymal or intracerebroventricular routes (Ma et al., 2013; Zheng et al., 2017; Zhao et al., 2018; Reza-Zaldivar et al., 2019). Delivering MSCs *via* the intrathecal route has also been identified as a less invasive delivery route, since it does not require brain surgery (Kim et al., 2020).

Focused ultrasound is another technology that has been leveraged to improve MSC homing (Liu et al., 2020). Application of focused ultrasound to the brain transiently ruptures the capillary lining of the BBB and supports delivery of therapeutic molecules into the brain through increased capillary permeability. Focused ultrasound application has also been shown to upregulate intercellular adhesion molecules (ICAMs), stromal cell-derived factor 1α (SDF-1α), monocyte chemotactic protein 1 (MCP-1), matrix metalloproteinase 9 (MMP9), and immune cell trophic factors that contribute to transient BBB opening and tropism of MSCs to the brain (Kovacs et al., 2017; Yang et al., 2019). Preclinical studies in rats have demonstrated that focused ultrasound to the hippocampus increases local expression of vascular cell adhesion molecule 1 (VCAM-1) and ICAM-1, and results in a more than 2-fold increase in the number of MSCs found in the sonicated region following intravenous infusion (Lee et al., 2020). Focused ultrasound has also been combined with contrast agents such as microbubbles to enhance BBB permeabilization. In response to ultrasound, microbubbles cavitate and locally exert various physical forces, a technique known as ultrasound-mediated microbubble destruction. In a rat model of brain ischemia, ultrasound-mediated microbubble destruction has been shown to increase the number of intravenously infused MSCs found in the brain parenchyma by 2.3-fold (Cui et al., 2020). The increased MSC homing appeared to correlate with greater recovery of neurological function, though the ultrasound-only control, which would be necessary to make this conclusion, was not included.

Magnetic targeting has also been investigated for improving MSC delivery to the brain. By labeling MSCs with superparamagnetic nanoparticles, they can be guided to the brain using external magnets. One such study found that magnetic targeting of intravenously infused MSCs improved their migration into the brains of AD rats, to levels comparable to those of intracerebroventricularly injected MSCs (Hour et al., 2020). Magnetic targeting also appeared to improve certain measures of cognitive function as well as expression of cholinergic signaling molecules.

### Genetic modification of mesenchymal stem/stromal cells

Studies have explored various genetic modifications of MSCs to enhancing their function or survival. For instance, MSCs overexpressing CX3CL1 have been shown to attenuate synaptic loss and the levels of pro-inflammatory cytokines in APP/PS1 transgenic AD mice (Li et al., 2020). While CX3CL1 overexpression alone did not lead to cognitive improvement, transplantation of MSCs overexpressing both CX3CL1 and Wnt3a successfully fostered hippocampal neurogenesis, improved cognitive function, and reduced microglia neurotoxicity (Li et al., 2020). In the same mouse model, MSCs overexpressing VEGF have been shown to improve hippocampal neovascularization, diminish senile plaques, inhibit inflammation, and rescue behavioral and cognitive functions as compared to control MSCs (Garcia et al., 2014).

Studies have revealed that microRNA-modified MSCs may play a beneficial role in treatment of neurodegenerative diseases like AD (Liu et al., 2015; Han et al., 2018). For instance, MSCs overexpressing BDNF have been shown to promote neuron survival when they are co-cultured with primary neurons isolated from APP/PS1 mice (Song et al., 2015). It was revealed that MSCs express low levels of Brn-4 protein despite the high expression of the transcription factor Brn-4 mRNA due to the presence of miR-937, which inhibits the translation of Brn-4 mRNA (Liu et al., 2015). However, bone marrow MSCs induced to express the antisense of miR-937 (as-miR-937) successfully suppressed miR-937 to increase expression of transcription factor Brn-4, which in turn increased BDNF protein levels and significantly enhanced the therapeutic effect of MSCs in the APP/PS1 mice (Liu et al., 2015). Additionally, MSCs overexpressing as-miR-937 had reduced Aβ accumulation, and enhanced cognitive function (Liu et al., 2015).

It has been observed that MSCs exposed to Aβ express early apoptosis and increased levels of the apoptosis mediating protease caspase 3, along with a decrease in levels of the microRNA let-7f-5p (Han et al., 2018). Upregulation of let-7f-5p countered the Aβ-induced apoptosis in MSCs by decreasing the levels of caspase-3, thereby prolonging MSC retention in the brain (Han et al., 2018). Elevation of let-7f-5p also reduced Aβ-induced cytotoxicity and enhanced survival of engrafted MSCs by targeting caspase-3 (Han et al., 2018).

Glucagon-like peptide-1 (GLP-1) has also been suggested to protect neurons from Aβ-mediated toxicity by preventing neuronal apoptosis, oxidative injury, and the generation and accumulation of Aβ deposits from APP (Perry and Greig, 2002). One study has shown that intracerebroventricular transplantation of GLP-1 overexpressing MSCs into AD mice reduced Aβ deposition and a downregulated microglial and astrocytic immunoreactivity in the brain (Klinge et al., 2011), though the reductions were moderate.

### Priming of mesenchymal stem/stromal cells

Priming MSCs with cytokines, hypoxia, nutrition deficiency, and various small molecules has been conducted to enhance the therapeutic efficacy and long-term survival of MSCs. For instance, pre-treating gingiva-derived MSCs (G-MSCs) with cannabidiol (CBD) modified the transcriptional profile of these MSCs to downregulate expression of proteins that are potentially involved in tau phosphorylation and Aβ production while upregulating genes involved in Aβ clearance and degradation (Libro et al., 2016). CBD-treated G-MSCs exhibited a downregulation of β- and γ-secretases which are usually responsible for Aβ production, and an upregulation of α-secretases which are responsible for the normal cleavage of APP (Libro et al., 2016). CBD treatment also upregulated the expressions of HSPs (HSP70s and HSP90s) and ubiquitin-conjugating enzymes, which enhances the clearance of aberrant proteins associated with AD pathology, such as Aβ plaques and neurofibrillary tangles. CBD was also found to bind to the vanilloid receptor 1 (TRPV1) to promote PI3K/Akt signaling and inhibit GSK3β, the latter of which is believed to be responsible for tau hyperphosphorylation. In another study, pre-treating MSCs with melatonin improved survival of transplanted adipose tissue-derived MSCs (ADMSCs) and better preserved the cognitive, learning, and memory functions in Aβ-treated AD rats (Nasiri et al., 2019). The number of activated microglia was also significantly decreased in melatonin-pre-treated ADMSCs group, and Aβ clearance was increased relative to untreated ADMSCs (Nasiri et al., 2019).

### Extracellular vesicles as cell-free therapy

The primary mechanism of action for MSC-based therapies is believed to be their paracrine activities *via* release of various secretory factors to facilitate tissue repair and immunomodulation. Due to this, many groups are investigating cell-free therapies based on MSC-derived EVs which have been shown to be sufficient for inhibiting apoptosis and reducing neuroinflammation (de Godoy et al., 2018; Kang et al., 2019). Conditioned media (CM) collected from different types of MSCs have been shown to reduce cognitive impairment in AD mice (Mita et al., 2015).

The use of MSC-derived EVs confers several advantages, including low immunogenicity, higher safety profile, ease of injection, and enhanced ability to trespass biological barriers. These attributes circumvent complications such as tumor formation, immune rejection, and undesired entrapment in the lung microvasculature. Moreover, MSC-derived EVs express the same set of membrane receptors and surface markers as MSCs, which may allow them to retain some of the homing capabilities possessed by their parent MSCs (Grange et al., 2014).

Mesenchymal stem/stromal cell-secreted EVs have demonstrated therapeutic efficacy in several AD studies. For instance, treating neural stem cells derived from the Tg2576 AD mouse model with ADMSC-derived EVs successfully reduced Aβ levels and neuronal apoptosis while fostering neuronal growth *in vitro* (Lee et al., 2018). It has been demonstrated that AD-MSC-derived EVs carry enzymatically active NEP and successfully degrade secreted and intracellular Aβ levels in Aβ-overexpressing neuroblastoma cells (Katsuda et al., 2013). Interestingly, the Aβ inhibition of these AD-MSC-derived EVs exceeded that of BM-MSCs (Katsuda et al., 2013). UC-MSC-derived EVs have also been shown to ameliorate cognitive dysfunction and neuroinflammation by modulating microglia activation in a mouse AD model (Ding et al., 2018).

Extracellular vesicles secreted from 3D-cultured MSCs seem to demonstrate higher therapeutic effect in several studies relative to those derived from 2D-cultured MSCs. 3D-cultured MSCs produced higher yield of EVs, and these EVs exhibited improved efficiency in delivering their contents to neurons (Haraszti et al., 2018). EVs secreted from UC-MSCs cultured in 3D graphene scaffold have been shown to differentially express hundreds of miRNAs compared to those collected from 2D-cultured UC-MSCs, and were enriched for several genes relevant to AD therapy such as HSP90, NEP, and IDE (Yang et al., 2020). These differences highlight the large variation in EV functional properties depending on the culture conditions, which will be a crucial factor to consider for successful clinical translation.

## Conclusion and future implications

Mesenchymal stem/stromal cells have been extensively investigated as a therapeutic strategy for treating neurodegenerative diseases such as AD. However, while preclinical studies have demonstrated some therapeutic potential of MSC-based therapies, several limitations mentioned above have hindered their effectiveness in clinical trials. Since intravenously administration of MSCs results in majority being trapped in the microvasculature of the lungs, finding a path of delivery that enables efficient MSC delivery and homing to the brain remains a challenge. In order to foster MSC migration to the brain and improve clinical therapeutic efficacy, studies have explored applications such as focused ultrasound, genetic modification, MSC conditioning, and local delivery of MSC into the brain. In addition to MSC-based cell therapy, cell free therapy based on MSC-derived EVs has also demonstrated potential in the treatment of AD.

As we have seen in this review, much of the preclinical literatures investigating MSCs for AD therapy have utilized widely different AD animal models, MSC sources, culture conditions and administration routes; while the reported therapeutic effects often attain statistical significance but are yet to be established in clinics. Nevertheless, the mechanistic understanding of how MSCs might ameliorate AD pathology still remains to be proven. The potential future of MSC-based therapy for AD hinges on a thorough scientific inquiry and mechanistic insight into the interaction between MSCs and neural cells, followed by rigorous preclinical studies that evaluate clinically meaningful endpoints. Majority of clinical trials have not lived up to their potential despite having good pre-clinical data due to limited consideration in pathological variability corresponding to different stages of the disease and its resulting impact on therapeutic effect of MSC-based therapies. Hence, well defined pre-clinical study protocols will be essential for effective clinical translation of novel therapeutics. Indeed, this can include testing specific animal models with therapeutic interventions mapped to specific stages of the disease. Furthermore, the actual therapeutic candidate should be controlled and evaluated for at the pre-clinical stage, in terms of its source, dose and route of administration. These specific parameters should then not be changed to ensure that the clinical translation is optimal less likely to fail. Finally, given that each patient will have their own unique physiology and microenvironment, modulation of the brain tissue at target sites may in fact help to ensure consistent and reproducible regenerative and protective responses. Without this groundwork, it is highly possible that clinical trials will continue to generate disappointing results.

## Author contributions

SR and AST contributed to conceptualization. SR, MS, and DDL contributed to writing the review. SR, MS, DDL, AG, RP, SC, RY, and AST contributed to editing and review. BDK contributed to preparing the figures. AST contributed to supervision. All authors contributed to the article and approved the submitted version.
